# Indomitable Perseverance

**Published:** 1864-12

**Authors:** 


### INDOMITABLE PERSEVERANCE.

In the history of the Cincinnati Observatory is one of the most remarkable instances on record of how much one man may accomplish without money and without means, simply by the force of the will when directed towards a laudable and possible enterprise. Had Professor Mitchell, who has the finest astronomical mind in the country, done less than he did do, the largest and best refracting telescope in the world but one would not have been placed on one of the hills of the Far West. Let his own simple unvarnished narration, as taken from his monthly *Sidereal Messenger*, be a lesson to all young men who want to achieve a great name:

On the 9th November, 1843, the corner-stone of the observatory was laid by John Quincy Adams, in the presence of a vast multitude, with appropriate ceremonies, and followed by the delivery of an address replete with beauty and eloquence. The season was too far advanced to permit any thing to be done towards the erection of the building during the Fall; and, indeed, it was not the intention of the Board of Directors to proceed with the building until every dollar required in the payment for the great telescope should have been remitted to Europe. At the time of laying the corner-stone but $3000 out of $9500 had been paid. This was the amount required in the contract to be paid on signing, and the remaining sum became due on finishing the instrument.

The contract having been made conditionally in July, 1842, it was believed that the great refractor would be shipped for the United States in June, 1844, and to meet our engagements the sum of $6500 must be raised.

This amount was subscribed, but in consequence of commercial difficulties, all efforts hitherto made to collect it had been unavailing, and in February, 1844, the Board of Control solicited the Director of the Observatory to become the general agent of the society, and to collect all old subscriptions, and obtain such new ones as might be necessary to make up the requisite sum. The accounts in the hands of the previous collector were accordingly turned over to me, and a systematic effort was made to close them up. A regular journal was kept of each day's work, noting the number of hours employed, the persons visited, those actually found, the sums collected, the promises to pay, the positive repudiations, the due bills taken, payable in cash and trade, and the day on which I was *requested to call again.* These intervals extended from a week or ten days to four months. The hour was in general fixed, and when the day rolled round, and the hour arrived, the agent of the society presented himself, and referred to the memoranda. In many cases another and another time was appointed, until, in some instances, almost as many calls were made as there were dollars due. By systematic perseverance, at the end of some forty days the sum of $3000 was paid over to the treasurer as the amount collected from old subscribers. Nearly $2000 of due bills had been taken, payable in carpenter's work, painting, dry goods, boots and shoes, hats and caps, plastering and bricklaying, blacksmith's work, paints and oils, groceries, pork barrels, flour, bacon and lard, hardware, iron, nails, etc., in short, in every variety of trade materials and workmanship. The due bills in cash brought about $500 in the course of the next thirty days, and a further sum of $3000 was required for the last remittance to Europe. It was determined to raise this amount in large sums from wealthy and liberal citizens who had already become members of our society. The list first made out, and the sums placed opposite the names of each person is now in my possession. On paper the exact amount was made up in the simplest and most expeditious manner; eight names had the sum of $200 opposite them, ten names were marked $100 each, and the remaining ones $50 each. Such was the singular accuracy in the calculation, that when the theory was reduced to practice, it failed in but one solitary instance. One person, upon whom we had relied for $200, declined absolutely, and his place was filled by another.

I called on one of the eight individuals marked at $200, and, after a few moments' conversation, he told me that in case $100 would be of any use to me, he would gladly subscribe that amount. I showed him my list, and finding his name among those reckoned at $200, he remarked that he would not mar so beautiful a scheme for the sum of $100, and accordingly entered his name in its appropriate place.

At a meeting held in May, by the Board of Control, the treasurer reported that the entire amount was now in the treasury with the exception of $150. The Board adjourned, to meet on the same day of the following week, when the deficiency was reduced by the agent to $25, and on the same day an order was passed to remit the entire amount to the Barings & Brothers, of London, to be paid to the manufacturer, on the order of W. J. Lamont, of Munich, to be given on the packing of the instrument. The last $25 was obtained and placed in the treasurer's hands immediately on the adjournment of the Board. Thus was completed, as it was supposed, by far the most difficult part of the enterprise. All the cash means of the society had now been exhausted; about $11,000 dollars had been raised, and to extend the effort yet further, under the circumstances, seemed to be quite impossible. Up to this time nothing had been done towards the building, and after paying for the instrument, not one dollar remained in cash to commence the erection of a building which must cost, at the lowest estimate, $5000 or $6000. Some $2000 or $3000 had been subscribed, payable in work and materials. Owing to a slight change in the plan of the building, the foundation walls already laid in the Fall of 1843 were taken up and relaid. Finding it quite impossible to induce any master-workman to take the contract for the building, with the many contingencies by which our affairs were surrounded, I determined to hire workmen by the day, and superintend the erection of the building personally. In attempting to contract for the delivery of brick on the summit of Mount Adams, such an enormous price was demanded for the hauling, in consequence of the steepness of the hill, that all idea of a brick building was at once abandoned, and it was determined to build of limestone, an abundance of which could be found on the grounds of the quarry. Having matured my plans, securing the occasional assistance of a carpenter, about the beginning of June, 1844, I hired two masons, one of whom was to receive an extra sum for hiring the hands, keeping their time, and acting as the master-workman. One tender to these workmen constituted the entire force with which I commenced the erection of the building, which, if prosecuted in the same humble manner, would have required about twenty years for its completion. And yet our title-bond required that the building should be finished in the following June, or a forfeiture of the title by which we hold the present beautiful site must follow. My master-mason seemed quite confounded when told that he must commence work with such a force. In the outset difficulties were thick and obstinate. Exorbitant charges were made for delivering lime. I at once commenced the building of a lime-kiln, and in a few days had the satisfaction of seeing it well filled, and on fire. True, it caved in once or twice, with other little accidents, but a full supply of lime was obtained, and at a cheap rate. Sand was the next item, for which the most extravagant charges were made. I found this so ruinous that an effort was made, and finally I obtained permission, to open a sand-pit, which had long been closed for fear of caving down a house, on the side of the hill above, by further excavation.

An absolute refusal was at first given, but systematic perseverance again succeeded, and the pit was re-opened. The distance was comparatively short, but the price of mere hauling was so great that I was forced to purchase horses, and in not a few instances to fill the carts with my own hands, and actually drive them to the top of the hill, thus demonstrating practically how many loads could be fairly made in a day. Another difficulty yet remained; no water could be found nearer than the foot of the hill, half a mile distant, and to haul the water so great a distance would have cost a large sum. I selected one of the deepest ravines on the hill-top, and throwing a dam across, while it was actually raining, I had the pleasure of seeing it fill rapidly from the hill-sides, and in this way an abundant supply was obtained for the mixing of mortar, at a very moderate expense of hauling.

Thus prepared, the building was commenced, with two masons and one tender during the first week; at the close of the week I had raised sufficient funds to pay off my hands, and directed the foreman to employ for the following week two additional masons and a tender; to supply this force with material, several hands were required in the quarry, in the lime-kiln, and in the sand-pit, all of whom were hired by the day, to be paid half cash, and the balance in trade. During all this time I may remark that I was discharging my duties as Professor of Mathematics and Philosophy in the Cincinnati College, and teaching five hours in each day. Before eight o'clock in the morning, I had visited all my workmen in the building, in the lime-kiln, sand-pit and stone quarry. At that hour my duties in the college commenced, and closed at one. By two o'clock, P. M., I was again with my workmen, or engaged in raising the means of paying them on Saturday night. The third week the number of hands was again doubled; the fourth week produced a like increase, until finally not less than fifty day laborers were actually engaged in the erection of the Cincinnati Observatory. Each Saturday night exhausted all my funds, but I commenced the next week in the full confidence that industry and perseverance would work out their legitimate results. To raise the cash means required was the great difficulty. I have frequently made four or five trades to turn my due bills, payable in trade, into cash. I have not unfrequently gone to individuals and sold them their own due bills payable in merchandise, for cash, by making a discount. The pork merchants paid me cash for my due bills, payable in barrels and lard kegs, and in this way I managed to obtain sufficient cash means to prosecute the work vigorously during the months of July and August, and in September I had the satisfaction to see the building up and covered, without having incurred one dollar of debt. At one period, I presume one hundred hands were employed at the same time in the prosecution of the work. More than fifty hands on the hill, and as many in the city, in the various workshops, paying their subscription by work for different parts of the building. The doors were in the hands of one carpenter, the window frames in those of another; a third was employed on the sash; a painter took them from the joiner, and in turn delivered them to a glazier, while a carpenter paid his stock by hanging them, with weights purchased by stock, and with cords obtained in the same way. Many locks were furnished by our own townsmen in payment of their subscriptions. Lumber, sawing, flooring, roofing, painting, mantels, steps, hearths, hardware, lathing, doors, windows, glass, and painting were in like manner obtained. At the beginning of each week my master carpenter generally gave me a bill of lumber and materials wanted during the week. In case they had not been already subscribed, the stock book was resorted to, and there was no relaxing of effort until the necessary articles were obtained. If a tier of joists were wanted, the saw mills were visited, and in some instances the joists for the same floor came from two or three different mills.

On covering the building, the great crowd of hands employed as masons, tenders, lime burners, quarry men, sand and water men, were paid off and discharged, and it now seemed that the heavy pressure was passed, and that we might again breathe free, after the responsibility of such heavy weekly payments was removed.

